# Supplementary material for: Deficiency of Complement Component C1Q Prevents Cerebrovascular Damage and White Matter Loss in a Mouse Model of Chronic Obesity
Source: eNeuro. 2020 Jun 2;7(3):ENEURO.0057-20.2020. doi: 10.1523/ENEURO.0057-20.2020 (PMC7294467; doi:10.1523/ENEURO.0057-20.2020)
Supplement: Extended Data Figure 1 — C1QA and C3 immunoreactivity in young WT and C1qa or C3 knockout mice. (A) C1QA (upper panel) and C3 (lower panel) immunoreactivity in the young WT corpus callosum is low or absence. (B) No C1QA immunoreactivity is found in the C1qa KO mice. (C) No C3 immunoreactivity is found in the C3 KO mice. Scale bars: 40μm. Download Extended Data Figure 1, DOCX file [file enu-eN-CFN-0057-20-s02.docx]

**Supplemental figures and legends**


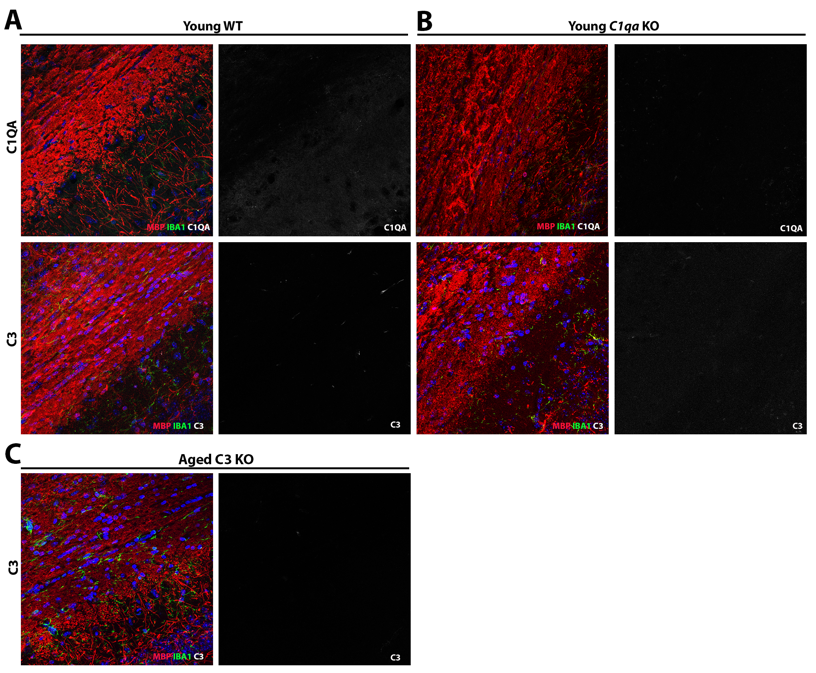


**Figure 1.** C1QA and C3 immunoreactivity in young WT and *C1qa* or *C3* knockout mice. (**A**) C1QA (upper panel) and C3 (lower panel) immunoreactivity in the young WT corpus callosum is low or absence. (**B**) No C1QA immunoreactivity is found in the *C1qa KO* mice. (**C**) No C3 immunoreactivity is found in the *C3 KO* mice. Scale bars: 40μm.

**Figure 2. Creation and validation of the *C1qa* KO mouse.** A conditional *C1qa* mouse was created as part of the Knockout Mouse Project (KOMP) at The Jackson Labs (https://www.jax.org/research-and-faculty/tools/knockout-mouse-project). (**A**) Representation of the *C1qa* alleles that are available including *C1qa^Tm1a^* (LacZ reporter), *C1qa^Tm1c^* (floxed allele), *C1qa^Tm1d^* (null allele). As has been shown for other KOMP alleles, the *Tm1a* allele is not capable of reporting *C1qa* expression using the β-galactosidase assay. Mating the *C1qa^Tm1a^* mice to mice carrying FLP recombinase creates the *C1qa^Tm1c^* floxed allele. Mating the *C1qa^Tm1c^* mice to mice carrying CRE recombinase creates the *C1qa^Tm1d^* null allele. Cell-specific ablation of *C1qa* can be achieved using a cell-specific Cre line. The locations of genotyping primers are shown as inward facing arrows. (**B**) Examples of genotyping assays for the different alleles and band sizes. Primers pairs in this example were as follows. *C1qa^+^*: F – CCGGAAGAAAAGACATCCTG; R – CTTTCACGCCCTTCAGTCCT. C1qa*^Tm1a^*: F – GTGGTTTGTCCAAACTCATCAA; R – TCTCTGAGCCTCTGCTTCAA. *C1qa^Tm1c^*: F – GGACGAGAGGGGAGGAGTTA; R – TTAGGACCCTTTGGCACAAC. *C1qa^Tm1d^*: F – CCGGAACCGAAGTTCCTATT; R – AGACGGGGATCGTTTATTCC. Note that the *C1qa^+^* primers amplify a larger product in mice carrying the *C1qa^Tm1c^* allele. Standard PCR conditions were used with an annealing temperature of 59.3^o^C for *C1qa^+^*, *C1qa^Tm1b^* and *C1qa^Tm1d^* and 61.0^o^C for *C1qa^Tm1c^*. (**C**) RNA in situ hybridization to visualize *C1qa* transcripts in brains sections from *C1qa^+/+^* and *C1qa^Tm^*

*^1d/Tm1d^* mice. As expected, *C1qa* expression is absent in *C1qa^Tm1d/Tm1d^* (*C1qa* KO) mice.
